# The Discovery of Oral Cancer Prognostic Factor Ranking Using Association Rule Mining

**DOI:** 10.1055/s-0043-1777050

**Published:** 2024-05-14

**Authors:** Sitthi Chaowchuen, Kritsasith Warin, Rachasak Somyanonthanakul, Wararit Panichkitkosolkul, Siriwan Suebnukarn

**Affiliations:** 1 Udonthani Cancer Hospital, Muang Udonthani, Udonthani, Thailand; 2 Faculty of Dentistry, Thammasat University, Pathum Thani, Thailand; 3 College of Digital Innovation Technology, Rangsit University, Pathum Thani, Thailand; 4 Faculty of Science and Technology, Thammasat University, Pathum Thani, Thailand

**Keywords:** oral cancer, prognostic factors, survival rate, data mining, association rule mining

## Abstract

**Objective** A 5-year survival rate is a predictor for the assessment of oral cancer prognosis. The purpose of this study is to analyze oral cancer data to discover and rank the prognostic factors associated with oral cancer 5-year survival using the association rule mining (ARM) technique.

**Materials and Methods** This study is a retrospective analysis of 897 oral cancer patients from a regional cancer center between 2011 and 2017. The 5-year survival rate was assessed. The multivariable Cox proportional hazards analysis was performed to determine prognostic factors. ARM was applied to clinicopathologic and treatment modalities data to identify and rank the prognostic factors associated with oral cancer 5-year survival.

**Results** The 5-year overall survival rate was 35.1%. Multivariable Cox proportional hazards analysis showed that tumor (T) stage, lymph node metastasis, surgical margin, extranodal extension, recurrence, and distant metastasis of tumor were significantly associated with overall survival rate ( *p* < 0.05). The top associated death within 5 years rule was positive extranodal extension, followed by positive perineural and lymphovascular invasion, with confidence levels of 0.808, 0.808, and 0.804, respectively.

**Conclusion** This study has shown that extranodal extension, and perineural and lymphovascular invasion were the top ranking and major deadly prognostic factors affecting the 5-year survival of oral cancer.

## Introduction

Oral cancer is the most common malignant tumor of the head and neck and is a highly malignant tumor with a relatively high mortality rate and a major health problem worldwide. The new cases and the number of deaths from oral cancer in 2020 were reported to be 1.8 million and 464,000 worldwide, respectively. 1 Oral cancer can be classified according to its origin (carcinoma and sarcoma). These neoplasms are aggressive in their biological behavior, leading to significant destruction of the structure of the oral cavity, and can develop local and distant metastases. 2 The gold standard for definitive diagnosis of oral cancer is confirmation by pathological examination. 3 4 Oral cancer treatment modalities depend on the American Joint Committee on Cancer (AJCC) tumor-node-metastasis (TNM) staging system, including tumor size, cervical lymph nodes, and distant metastases. 5 The main therapeutic approach for oral cancer over the past decade has not changed; it is surgical treatment followed by adjuvant radiation therapy with/without chemotherapy in cases with high-risk pathologic features or late-stage oral cancer. 3 6 7 In addition, oral cancer has a critical influence on patients in terms of facial appearance after treatment, ability to perform daily activities, ability to work, and quality of life. 8 9 Nevertheless, improvements in medical imaging, surgical and adjuvant chemoradiotherapy techniques, and advances in supportive care modalities may improve the quality of life, but not significantly improve the 5-year survival rate of oral cancer patients. 10 Thus, analyzing oral cancer data with data mining techniques to extract patterns between clinicopathologic factors, treatment, and 5-year survival outcome could provide an opportunity to better understand the pattern of oral cancer prognosis.

Data mining, known as knowledge discovery from data, is the process of extracting potentially useful information and identifying knowledge hidden in a large amount of data. Unlike traditional statistical research methods, data mining technologies mine information to discover knowledge based on unclear assumptions. 11 12 In the medical field, data mining techniques have the potential to capture complex details and patterns in medical data to predict disease. 13 For example, using time-series analysis and association rule mining (ARM) model to predict the number of Coronavirus Disease-2019 cases. 14 ARM is a pattern-extracted data mining technique, which was first introduced by Agrawal et al. as a method of analyzing marketing data. ARM consists of two steps: the first is to identify the frequent itemsets from the data, and the second is to generate the association rules from the frequent itemsets. 15 ARM has a different concept from conventional statistics, that is, ARM is the process of deriving useful insights and being able to extract meaningful patterns from the data, while conventional statistics is the science of collection, analysis, and interpretation of data. 16 The ARM technique is considered a useful tool in the medical field to provide the ability to perform intelligent diagnoses, extract invaluable information, and automatically create important insights while identifying relationships within and between interested variables. 16 An ARM is utilized to mine cancer data from a medical record to extract the significant pattern to discover the most common factors related to cancer biology and clinical prognosis. For example, ARM was utilized to decode molecular mechanisms of renal cell carcinoma subtypes 17 and to predict breast cancer recurrence. 18 In addition, previous studies have applied ARM to extract history and clinical data of oral cancer to discover the pattern for early detection and prevention of oral cancer. 19 20 Therefore, utilizing the ARM technique to extract the remarkable pattern of relationship between clinicopathologic, treatment, and 5-year survival data of oral cancer could be beneficial to aid clinicians' decision-making in oral cancer treatment.

The aim of this study is to analyze oral cancer data, including clinicopathologic features, treatment, and 5-year overall survival data, to discover and rank the prognostic factors associated with oral cancer 5-year survival using the ARM technique. The main contribution of this work is to offer an alternative analytical methodology, including conventional statistics, to define new, useful, and interesting relationships between various cancer factors and survival outcomes of oral cancer. This work is expected to provide supplementary information for aiding clinicians' decision-making in clinical practice.

## Materials and Methods

### Data Acquisition

This study was approved by the Human Research Ethics Committee of Thammasat University (COE 015/2565) and was performed in accordance with the tenets of the Declaration of Helsinki. Informed consent was waived by the Human Research Ethics Committee of Thammasat University because of the retrospective nature of the fully anonymized data.

**Fig. 1:**
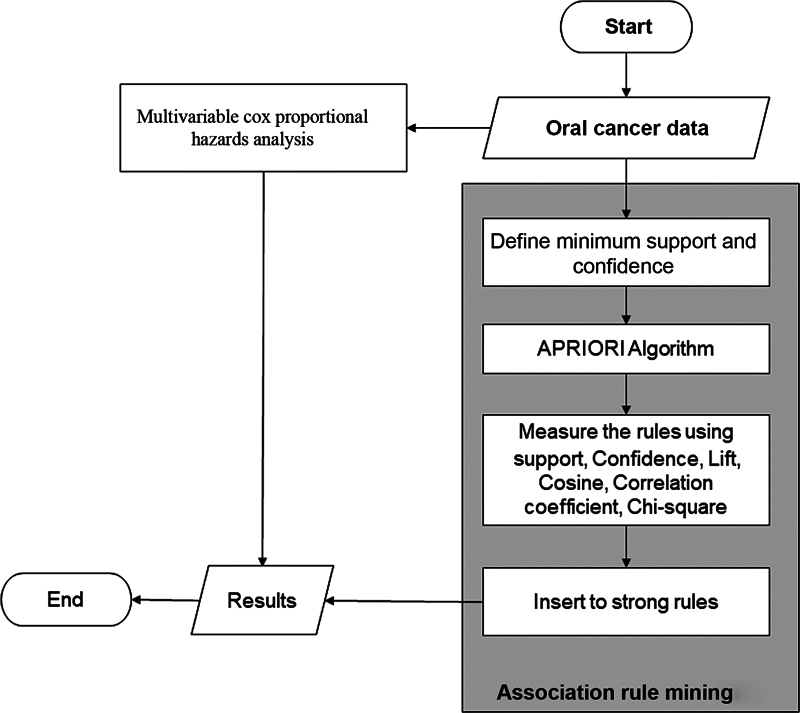
The methodology flowchart.

Oral cancer data were collected from electronic medical records from a regional cancer center of Thailand between 2011 and 2017. All cases of oral cancer were diagnosed by pathological examination as the gold standard of oral cancer diagnosis and follow-up for at least 5 years. In this study, oral cancer staging is according to the TNM staging classification system as proposed by the eighth edition AJCC cancer staging of head and neck cancer. 21 In addition, patients with pathological results of carcinoma *in situ* and with cancer in areas other than the oral cavity were excluded. Selection was based on completeness of clinicopathologic data, treatment modalities, and 5-year overall survivability data. After deleting cases with incomplete data, a total of 897 oral cancer cases, including squamous cell carcinoma (SCC), undifferentiated carcinoma, nonkeratinizing carcinoma, adenoid cystic carcinoma, mucoepidermoid carcinoma, and other types of oral cancer, remained available for analysis. The workflow of this study is illustrated in Fig. 1 .

### Statistical Analysis

Three types of data are available for analysis: (1) clinicopathological data (gender, age range, comorbidities, tumor location, clinical tumor [T] stage, cervical lymph node [N] stage, which is the pathological lymph node stage in surgical cases and the clinical lymph node stage in nonsurgical cases, distant metastasis [M], TNM stage, tumor types, lymph node metastasis, surgical margin, extranodal extension, lymphovascular invasion, perineural invasion, and recurrence); (2) treatment modalities data (surgery only, surgery with radiotherapy, surgery and concurrent chemoradiotherapy, induction chemotherapy, concurrent chemoradiotherapy, and palliative treatment); and (3) 5-year survivability data. Descriptive statistics were calculated for clinicopathological, treatment modalities, and survivability data. The overall and each TNM stage of the 5-year survival rate were calculated. The multivariable Cox proportional hazards model was used to determine independent predictors of 5-year survival rate and was performed with binary logistic regression, which included clinicopathological, treatment modalities, and survivability data. In addition, the Kaplan–Meier method was calculated to determine the cumulative proportion surviving and to plot the tumor stage survival curves. The data were analyzed using IBM SPSS Statistics version 26.

### Association Rule Mining (ARM)

ARM discovers the pattern of frequent items or events in the dataset, including the association between items and events. The pattern exposes the combination of the items or events that occur at the same time. In the medical field, it is helpful to know how one disease is associated with others. ARM can be used as a multivariate analysis of the correlation between factors. Given a dataset containing a collection of records or transactions, each record comprises a set of categorical attributes. An association rule can be denoted by *A → B* , where *A* (the antecedent or left-hand side [LHS]) and *B* (the consequent or right-hand side [RHS]) are sets of various attribute–value pairs (also called itemset) and are separate. 14 22 23 Generally, the effectiveness of discovered rules is measured in terms of Support, Confidence, and Lift. The rule represents the assumption that when variables in *A* occur in the dataset, the variables in *B* also occur. Association mining generates a large number of rules from a given dataset. The goal of this approach is to find rules that have high practical significance. To eliminate false rules, the effectiveness of discovered rules is measured in terms of Support, Confidence, Lift, Cosine, and Correlation coefficient. In addition, this study also uses the chi-squared test to measure the statistical significance of the association between the antecedent and the consequent. 14 22 23 24 25

Support refers to the number of records where the attribute–value pairs in either set *A* or *B* appear in the dataset relative to the total number of records (transactions or instances), which indicates how frequently the itemset appears in the dataset. The Support value is symmetric so that Support ( *A → B* ) = Support ( *B → A* ), and it equals the total numbers of records containing both *A* and *B* to the total number of records in the dataset.

The Confidence of the rule *A → B* measures the conditional probability of *B* , given A, which determines how frequently *B* appears in those who have *A* . Therefore, the Confidence measure for a given rule is asymmetric, that is, Confidence ( *A → B* ) ≠ Confidence ( *B → A* ). The confidence is the conditional probability of occurrence of consequent given the antecedent. However, the value of confidence limits our capability to make an inference.

The Lift measure is the ratio between the observed support and the expected support between the independent variables *A* and *B* . The lift suggests how often *B* appears when *A* appears while controlling the likely occurrence of *B* . The value of lift determines the correlation between *A* and *B* : lift = 1 indicates independence, lift more than 1 indicates positive relationship, and lift less than 1 indicates negative relationship. Lift is also a symmetric measure between the itemset A and B, i.e., Lift ( *A → B* ) = Lift ( *B → A* ). More the value of lift, greater are the chances of preference to consequent if the antecedent has already occurred. Lift is a measure that shows an importance of small item. If the lift is more than 1, these rules are potentially useful for predicting consequences in future datasets. 26

Cosine measures organize and summarize correlations based on “similarity,” which will provide a consistent and accurate view of correlations. The Cosine measure for the two rules can be organized into binary-valued vectors. It will give a value of 0 or 1 depending on whether the common between the two rules is present on the RHS or LHS of the rule; generally, a value higher than 0.5 shows strong similarities. 27 Cosine measures the similarity between two vectors of an inner product space that refers to distance with dimensions representing features of the data object, in a dataset.

Correlation coefficient measures the strength of the linear relationship between a pair of two variables. A high correlation points to a strong relationship between the two variables, while a low correlation means that the variables are weakly related. 14 22 23 24 25

A chi-squared test is used in the analysis of contingency tables when the sample sizes are large. It is primarily used to examine whether two categorical variables are independent in influencing the test. Chi-squared test is used to determine whether there is a statistically significant difference between the expected frequencies and the observed frequencies in one or more categories of a contingency table.

The formulation of Support, Confidence, Lift, Cosine, Correlation coefficient, and Chi-square was calculated as follows:

| *A* | and | *B* | are the numbers of records that include *A* and *B* .
| *A* ⋂ *B* ∣ is the number of records that contain both *A* and *B* .
*N* is the total number of patients.

In this study, ARM was implemented by a Python script and applied to clinicopathologic treatment modalities data to identify the survivability rules. The redundant rules were filtered, and significant rules identified. The antecedent *A* corresponds to clinicopathologic factors (gender, age range, comorbidities, tumor location, T stage, pathologic N stage, TNM stage, tumor types, lymph node metastasis, surgical margin, extranodal extension, lymphovascular invasion, perineural invasion, recurrence, and distant metastasis), and treatment modalities (surgery only, surgery with radiotherapy, surgery and concurrent chemoradiotherapy, induction chemotherapy, concurrent chemoradiotherapy, and palliative treatment). Furthermore, the consequent *B* focuses on 5-year survivability, including (1) death within 5 years and (2) survival of more than 5 years. Since one assumption for ARM is that all the values of attributes are discrete, the numerical data used in the study were translated into discrete labels.

## Results

The data characteristics of 897 oral cancer cases included in the study are shown in Table 1 . There were 460 males (51.3%) and 437 females (48.7%), and the mean age was 64 years (standard deviation 8.88 years). The preoperative TNM stages were: 91 cases (10.1%) of stage I, 135 cases (15.1%) of stage II, 135 cases (15.1%) of stage III, 481 cases (53.6%) of stage IVa, 48 cases (5.4%) of stage IVb, and 7 cases (0.8%) of stage IVc. The most common tumor location was oral tongue, which accounted for 405 cases (45.2%). The most common tumor types were SCC, which accounted for 797 cases (88.9%). The treatment modalities comprised surgery only of 146 cases (16.3%), surgery with radiotherapy of 249 cases (27.8%), surgery and concurrent chemoradiotherapy of 126 cases (14.0%), induction chemotherapy of 60 cases (6.7%), concurrent chemoradiotherapy of 222 cases (24.7%), and palliative treatment of 94 cases (10.5%). The survival data of patients were recorded in survival length, including 1 to 12 months of 367 cases (40.9%), 13 to 24 months of 133 cases (14.8%), 25 to 36 months of 46 cases (5.1%), 37 to 48 months of 26 cases (2.9%), 49 to 60 months of 10 cases (1.1%), and more than 60 months or 5 years of 315 cases (35.1%).

**Table 1:** Summary of clinicopathologic, treatment modalities, and survival data of the oral cancer patients

Variable		Number ( *n* = 897)	Percentage (%)
Gender	Male	460	51.3
	Female	437	48.7
Mean age in years (SD)		64 (8.88)	
Comorbidities	None	565	74.9
	Diabetes mellitus	48	5.4
	Hypertension	90	10.0
	Dyslipidemia	4	0.4
	Heart disease	7	0.8
	Neurological disease	5	0.6
	Kidney disease	9	1.0
	Multiple diseases	60	6.7
	Others	2	0.2
Tumor location	Oral tongue	405	45.2
	Floor of mouth	112	12.5
	Buccal mucosa	108	12.0
	Alveolar ridge	120	13.4
	Hard palate	37	4.1
	Lip	78	8.7
	Retromolar trigone	33	3.7
	Uncertain site	4	0.4
T stage	T1	125	13.9
	T2	240	26.8
	T3	128	14.3
	T4a	370	41.2
	T4b	34	3.8
N stage	N0	407	45.4
	N1	150	16.7
	N2a	70	7.8
	N2b	167	18.6
	N2c	83	9.3
	N3	20	2.2
TNM stage	Stage I	91	10.1
	Stage II	135	15.1
	Stage III	135	15.1
	Stage IVa	481	53.6
	Stage IVb	48	5.4
	Stage IVc	7	0.8
Tumor types	SCC, WD	522	58.2
	SCC, MD	234	26.1
	SCC, PD	41	4.6
	Undifferentiated CA	18	2.0
	Nonkeratinizing CA	9	1.0
	Adenoid cystic CA	29	3.2
	Mucoepidermoid CA	18	2.0
	Others	26	2.9
Lymph node metastasis	Negative	407	45.4
	Positive	490	54.6
Surgical margin	Negative	397	44.3
	Positive	133	14.8
	N/A a	367	40.9
Extranodal extension	Negative	473	52.7
	Positive	46	5.1
	N/A a	378	42.1
Lymphovascular invasion	Negative	473	48.7
	Positive	93	10.4
	N/A a	367	40.9
Perineural invasion	Negative	437	48.7
	Positive	93	10.4
	N/A a	367	40.9
Recurrence	No	705	78.6
	Local recurrence	81	9.0
	Regional recurrence	49	5.5
	Locoregional recurrence	62	6.9
Distant metastasis	No	832	92.8
	Lung metastasis	49	5.5
	Bone metastasis	11	1.2
	Brain metastasis	1	0.1
	Other area metastasis	4	0.4
Treatment modalities	Surgery only	149	16.6
	Surgery and radiotherapy	252	28.1
	Surgery and concurrent chemoradiotherapy	129	14.3
	Induction chemotherapy	57	6.4
	Concurrent chemoradiotherapy	216	24.1
	Palliative treatment	94	10.5
Survival length	1–12 months	367	40.9
	13–24 months	133	14.8
	25–36 months	46	5.1
	37–48 months	26	2.9
	49–60 months	10	1.1
	>60 months	315	35.1

Abbreviations: CA, carcinoma; MD, moderately differentiate; PD, poorly differentiate; SCC, squamous cell carcinoma; SD, standard deviation; TNM, tumor-node-metastasis; WD well differentiate.

a N/A is a nonavailable data in the nonsurgical group or uninterpreted data in the pathological record.

### Five-Year Survival Rate

**Fig. 2:**
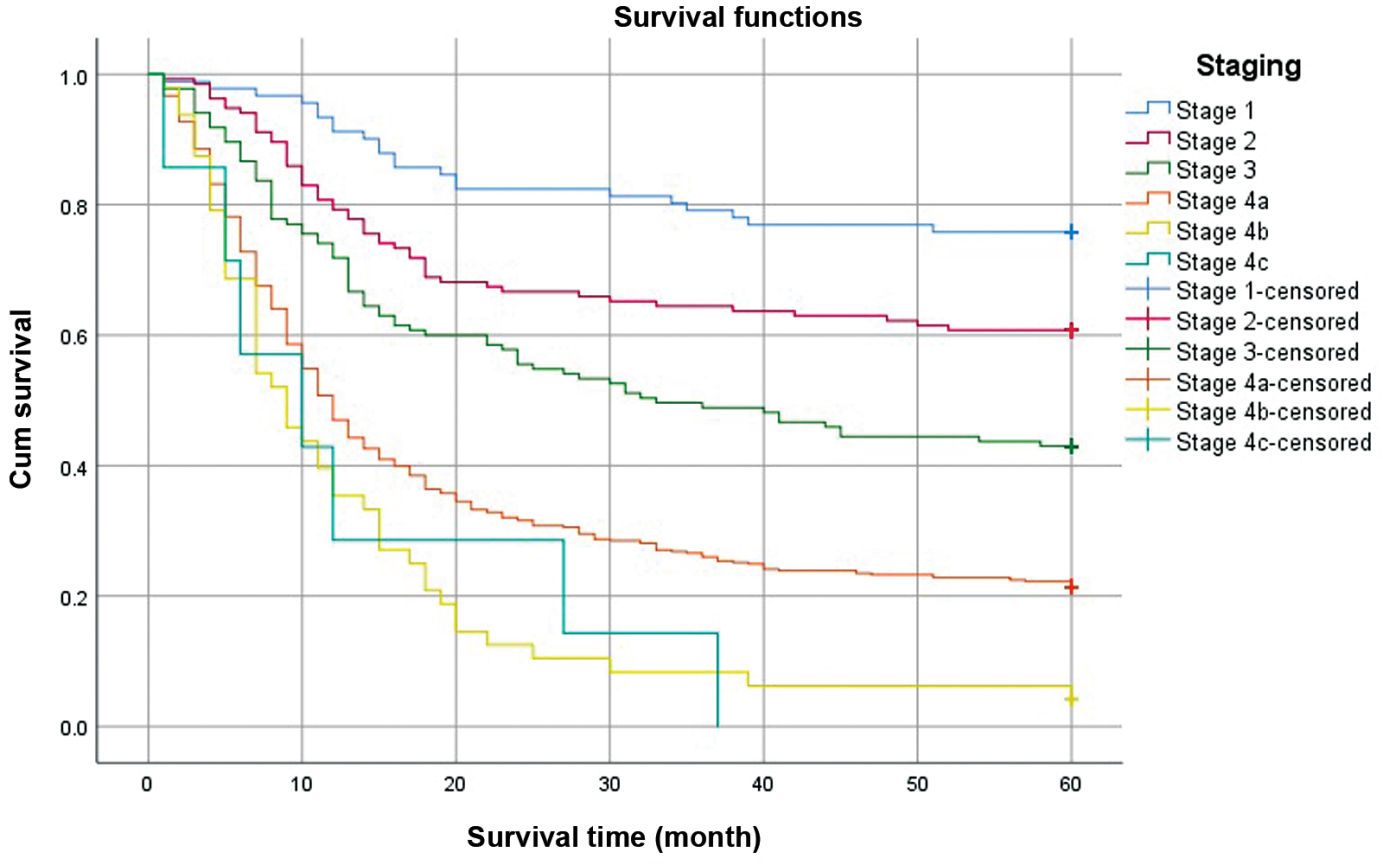
Kaplan–Meier survival curves of 5-year survival rate by tumor-node-metastasis stage of oral cancer: Y-axis, Cumulative survival; X-axis, Survival time (month).

The 5-year overall survival rate in this study was 35.1%, which was divided into stage I of 75.8%, stage II of 60.7%, stage III of 43.0%, stage IVa of 21.4%, stage IVb of 4.2% and stage IVc of 0%. The Kaplan–Meier curves of the 5-year survival rate of TNM stage are shown in Fig. 2 .

### Multivariable Cox's Proportional Hazards Analysis

In the multivariate analysis, this study found that T stage, positive lymph node metastasis, surgical margin, extranodal extension, lymphovascular invasion, recurrence of tumor, and presence of distant metastasis were significantly correlated to overall 5-year survival rate ( *p* < 0.05) ( Table 2 ).

**Table 2:** Multivariate Cox proportional hazard analysis for overall 5-year survival ( *n* = 897; 582 death events)

Variables		Hazard ratio	95% CI	*p* -Value
T stage	T1	1	–	–
	T2	2.151	1.325–3.492	0.002 a
	T3	2.926	1.743–4.914	0.000 a
	T4a	3.460	2.142–5.590	0.000 a
	T4b	3.655	1.017–13.136	0.047 a
Lymph node metastasis	Negative	1	–	–
	Positive	1.716	1.291–2.281	0.000 a
Surgical margin	Negative	1	–	–
	Positive	1.449	1.090–1.926	0.011 a
Extranodal extension	Negative	1	–	–
	Positive	1.780	1.172–2.702	0.007 a
Lymphovascular invasion	Negative		–	–
	Positive	1.800	1.340–2.416	0.000 a
Recurrence	No recurrence	1	–	–
	Local recurrence	2.426	1.684–3.494	0.000 a
	Regional recurrence	3.029	2.029–4.522	0.000 a
	Locoregional recurrence	5.003	3.186–7.855	0.000 a
Distant metastasis	No metastasis	1	–	–
	Metastasis	2.360	1.498–3.716	0.000 a

Abbreviation: CI, confidence interval.

a *p* -Value < 0.05.

### Five-Year Survivability Association Rules

ARM was applied to identify the rules associated to 5-year survivability of oral cancer, including death within 5 years and survival of more than 5 years. The top 10 death within 5 years rules ranked by highest confidence scores are presented in Table 3 . Among the top 10 rules, positive extranodal extension (Support of 0.274, Confidence of 0.808, Lift of 1.552, Cosine of 0.669, Correlation coefficient of 0.412, and Chi-square of 135.788) was the major rule of death within 5 years, followed by positive perineural invasion and positive lymphovascular invasion. The top 10 survival for more than 5 years rules ranked by highest confidence scores are presented in Table 3 . Among the top 10 rules, patient with negative lymphovascular invasion (Support of 0.381, Confidence of 0.576, Lift of 1.390, Cosine of 0.727, Correlation coefficient of 0.458, and Chi-square of 167.31) was the major rule of survival greater than 5 years, followed by negative perineural invasion and negative extranodal extension.

**Table 3:** Top 10 Association Rule of 5-year survivability ranked by confidence (death within 5 years and survive more than 5 years)

Rule	Antecedent (LHS)	Consequent (RHS)	Support	Confidence	Lift	Cosine	Correlation coefficient	Chi-square	OR (95% CI)
1	Extranodal extension: positive	Death within 5 years	0.274	0.808	1.552	0.669	0.412	135.788	4.149 (2.004–8.621)
2	Perineural invasion: positive	Death within 5 years	0.274	0.808	1.651	0.670	0.457	166.746	5.401 (2.915–10.0)
3	Lymphovascular invasion: positive	Death within 5 years	0.273	0.804	1.644	0.670	0.452	79.026	5.747 (3.106–10.638)
4	Surgical margin: positive	Death within 5 years	0.262	0.771	1.688	0.665	0.452	0.0037	5.682 (3.236–10.0)
5	Tumor types: SCC, MD	Death within 5 years	0.232	0.683	1.045	0.492	0.044	1.559	1.629 (0.485–5.479)
6	pN stage: pN2a	Death within 5 years	0.228	0.672	1.482	0.581	0.315	79.026	2.295 (1.481–3.558)
7	Comorbidities: diabetes mellitus	Death within 5 years	0.215	0.635	0.989	0.461	–0.011	0.0968	1.715 (0.811–3.623)
8	Gender: male	Death within 5 years	0.178	0.524	1.087	0.440	0.060	2.916	0.774 (0.578–1.038)
9	Gender: female	Death within 5 years	0.161	0.476	0.919	0.385	–0.060	2.916	0.774 (0.578–1.038)
10	T stage: T4a	Death within 5 years	0.130	0.384	1.503	0.442	0.211	48.287	14.085 (12.195–100.0)
1	Lymphovascular invasion: negative	Survive more than 5 years	0.381	0.576	1.390	0.727	0.458	167.31	0.07 (0.045–0.11)
2	Perineural invasion: negative	Survive more than 5 years	0.381	0.576	1.390	0.727	0.458	138.757	0.07 (0.045–0.11)
3	Extranodal extension: negative	Survive more than 5 years	0.381	0.576	1.345	0.715	0.417	167.31	0.112 (0.076–0.166)
4	Surgical margin: N/A	Survive more than 5 years	0.379	0.574	1.389	0.726	0.456	166.129	0.067 (0.043–0.104)
5	Gender: female	Survive more than 5 years	0.357	0.540	1.042	0.610	0.060	2.916	1.291 (0.963–1.731)
6	T stage: T4b	Survive more than 5 years	0.353	0.534	1.301	0.678	0.3501	98.241	0.192 (0.026–1.436)
7	Tumor location: buccal mucosa	Survive more than 5 years	0.327	0.494	1.021	0.577	0.028	0.619	1.276 (0.815–1.998)
8	Gender: male	Survive more than 5 years	0.304	0.460	0.955	0.539	–0.060	2.916	1.291 (0.963–1.731)
9	Lymph node metastasis: negative	Survive more than 5 years	0.225	0.341	0.752	0.412	–0.314	79.026	0.253 (0.345–0.185)
10	Surgical margin: negative	Survive more than 5 years	0.086	0.131	1.004	0.294	0.002	0.004	0.07 (0.043–0.104)

Abbreviations: CI, confidence interval; LHS, left-hand side; MD, moderately differentiate; N/A, nonavailable; OR, odds ratio; RHS, right-hand side; SCC, squamous cell carcinoma.

## Discussion

This work examined the effectiveness of ARM in extracting a set of meaningful rules to determine remarkable prognostic factors of oral cancer using clinicopathologic, treatment modalities, and 5-year survivability data. The 5-year survival rate of oral cancer is a key indicator of prognosis and treatment success, and understanding the factors related to this survival rate is important to improve patient prognosis. In this study, the overall 5-year survival rate of oral cancer was 35%, which was relatively lower than previous studies that reported an overall 5-year survival rate of 76 to 83.3%. 28 29 30 31 The low 5-year survival rate could stem from the fact that most patients in this study were diagnosed at an advanced stage, which had a poor prognosis with a 5-year survival rate of 30%, 2.5%, and 0% for stages IVa, IVb, and IVc, respectively. Prognostic factors analyzed by the multivariable Cox proportional hazards analysis of this study revealed that T stage, positive lymph node metastases, surgical margin, extranodal extension, lymphovascular invasion, tumor recurrence, and presence of distant metastases were significantly affected by the overall 5-year survival rate of oral cancer ( *p* < 0.05), which was similar to previous studies that found adverse pathologic features, tumor recurrence, and distant metastasis correlated with 5-year survival rates of oral cancer. 28 32 33 34 Although the multivariable analysis could identify the significant prognostic factors related to 5-year survival rate of oral cancer, it could not contribute to the ranking of significant factors. Therefore, the application of a computational technique, the ARM technique, to oral cancer data could extract and provide new information by ranking the prognostic patterns of oral cancer for additional insights for clinicians' decision-making in the clinical practice.

In the ARM analysis of oral cancer data, the top five deaths within the 5 years rules of oral cancer included a positive extranodal extension, perineural invasion, lymphovascular invasion, surgical margin, and tumor type of SCC with moderately differentiate (MD) with a Support of 0.232 to 0.274, Confidence of 0.683 to 0.808, Lift of 1.045 to 1.688, Cosine of 0.492 to 0.67, and Correlation coefficient of 0.044 to 0.457. Furthermore, the top five survival rules consisted of patient with negative lymphovascular invasion, perineural invasion, extranodal extension, and nonavailable data of surgical margin group and female patients with a Support of 0.357 to 0.381, Confidence of 0.574 to 0.576, Lift of 1.042 to 1.390, Cosine of 0.61 to 0.727, and Correlation coefficient of 0.006 to 0.458. These meant that if a patient exhibited positive extranodal extension, perineural or lymphovascular invasion, surgical margin, and SCC with MD, then there was higher confidence of that oral cancer patient dying within 5 years. The results of ARM corresponded with the multivariate analysis, which found that the extranodal extension, lymphovascular invasion, and surgical margin significantly impacted the 5-year survival rate of oral cancer. Nevertheless, the ARM contributed to the ranking of these factors, which showed that positive extranodal extension was the top ranking related to 5-year survival rate of oral cancer. These results of ARM were an additional information to emphasize that the presence of these adverse pathologic features was the major deadly prognostic pattern for oral cancer, which the clinicians should focus on and be concerned about.

As per our understanding, this is the first study conducted to date to define death within 5 years and survive more than 5 years rules in oral cancer using ARM techniques, which prevents this study from comparing these findings with those of other studies. In addition, some exploratory and review studies have reported the strong relationship between various factors including advanced age, T stage, N stage, TNM stage, adverse pathologic features, tumor types, local and regional recurrence, and distant metastases, with oral cancer survival rate. 28 33 34 35 However, previous studies that analyzed the data with traditional statistics could only provide the significant factors but could not contribute to the ranking of prognostic factors related to oral cancer survivability. Therefore, these results provide new insights for the exploration of prognostic factors and reveal invaluable information about the deadly pattern of oral cancer. This work has theoretical and practical importance, which can serve as a reference for relevant studies in the future and will aid clinicians as supplementary information to predict oral cancer prognosis and select the most appropriate treatment plan for oral cancer patients.

The limitation of this study needs to be addressed. First, rule mining analysis is primarily for exploring associations and patterns in data. One of the main challenges of ARM is the ability to generate an overwhelming number of rules from a large dataset, which can be costly and complex to analyze. Second, the depth of invasion, which is a pathologic feature and important prognostic factor of oral cancer, was not included and analyzed due to the missing data in the medical and pathologic record. As the previous edition of AJCC cancer staging of head and neck cancer did not mention the depth of invasion for the cancer staging, 36 so there is no record in this cancer center between 2011 and 2017. Therefore, future research should build on this methodology linked with online data sources to collect more oral cancer data, including medical, radiologic, pathologic, and genomic data, from various cancer and health centers to achieve completed oral cancer data so that a decent number of meaningful prognostic rules can be extracted. In addition, the combination of artificial intelligence technology and analysis with different data mining techniques, including causal inference, could extract and provide other significant information about factors related to survival rate for predicting the oral cancer prognosis in the clinical practice. Furthermore, the application of machine learning techniques, including decision tree and deep learning algorithm, to create a prognostic prediction model, could be combined with ARM to create a prognostic prediction model with multiple variables allowing to establish a survival prediction for oral cancer cases to be applied in a real clinical scenario.

## Conclusion

Introducing the ARM technique into oral cancer data as a powerful approach can extract and classify data to uncover an interesting relationship between prognostic factors and the 5-year survival rate of oral cancer. The ARM identified the major ranking of the deadly prognostic rules of oral cancer, which were extranodal extension, perineural invasion, and lymphovascular invasion. The results of this study will provide important insights into the pattern and ranking of prognostic factors that influence the 5-year survival rate of oral cancer and may aid clinicians in selecting the most appropriate treatment plan to increase the survival rate for oral cancer patients.
